# The Cell Wall Deacetylases Spy1094 and Spy1370 Contribute to *Streptococcus pyogenes* Virulence

**DOI:** 10.3390/microorganisms11020305

**Published:** 2023-01-24

**Authors:** Tiger Aspell, Adrina Hema J. Khemlani, Catherine Jia-Yun Tsai, Jacelyn Mei San Loh, Thomas Proft

**Affiliations:** 1 Department of Molecular Medicine & Pathology, School of Medical Sciences, The University of Auckland, Private Bag 92019, Auckland 1142, New Zealand; 2 Maurice Wilkins Centre for Molecular Biodiscovery, The University of Auckland, Private Bag 92019, Auckland 1142, New Zealand

**Keywords:** *Streptococcus pyogenes*, group A Streptococcus, deacetylase, lysozyme resistance, gene deletion mutants, *Lactococcus lactis*, biofilm, whole blood killing assay, *Galleria mellonella* infection model

## Abstract

*Streptococcus pyogenes*, or Group A Streptococcus (GAS), is a strictly human pathogen that causes a wide range of diseases, including skin and soft tissue infections, toxic shock syndrome and acute rheumatic fever. We have recently reported that Spy1094 and Spy1370 of *S. pyogenes* serotype M1 are N-acetylglucosamine (GlcNAc) deacetylases. We have generated *spy1094* and *spy1370* gene deletion mutants in *S. pyogenes* and gain-of-function mutants in *Lactococcus lactis*. Similar to other cell wall deacetylases, our results show that Spy1094 and Spy1370 confer lysozyme-resistance. Furthermore, deletion of the genes decreased *S. pyogenes* virulence in a human whole blood killing assay and a *Galleria mellonella* (Greater wax moth) larvae infection model. Expression of the two genes in *L. lactis* resulted in increased lysozyme resistance and survival in whole human blood, and reduced survival of infected *G. mellonella* larvae. Deletion of the *spy1370*, but not the *spy1094* gene, decreased resistance to the cationic antimicrobial peptide cecropin B, whereas both enzymes increased biofilm formation, probably resulting from the increase in positive charges due to deacetylation of the cell wall. In conclusion, Spy1094 and Spy1370 are important *S. pyogenes* virulence factors and might represent attractive targets for the development of antibacterial agents.

## 1. Introduction

*Streptococcus pyogenes*, or Group A Streptococcus (GAS), is an exclusively human pathogen that can cause a wide range of diseases. These include common skin and soft tissue infections such as pharyngitis, tonsillitis, impetigo, erysipelas and cellulitis [1,2]. Severe invasive GAS diseases such as necrotising fasciitis (‘flesh-eating disease’) and streptococcal toxic shock syndrome are less common but have high mortality rates (30–70%) [3,4,5,6]. Untreated cases of pharyngitis and skin infections can result in post-streptococcal autoimmune diseases such as acute rheumatic fever, rheumatic heart disease and acute glomerulonephritis [7,8,9]. It is estimated that *S. pyogenes* is responsible for approximately 500,000 deaths, globally, each year [10]. 

The success of *S. pyogenes* as a major human pathogen can be attributed to the production of a large arsenal of virulence factors, which include adhesins [11,12], pili [13], superantigens [14], cytolysins [15], fibrinolysin [16] and complement evasion factors [17]. Another virulence strategy of some bacterial pathogens involves the deacetylation of peptidoglycan in the cell wall which is made up of alternating β-1,4-linked N-acetylglucosamine (GlcNAc) and N-acetylmuramic acid (MurNAc) residues cross-linked by a short peptide [18]. Deacetylation of the peptidoglycan cell wall results in decreased susceptibility to lysozyme, a muramidase that cleaves the amide bond between GlcNAc and MurNAc [19]. Lysozyme is a naturally occurring antimicrobial agent found in bodily secretions such as tears, saliva and milk. Lysosomal enzymes are also found in human phagocytes and assist in bacterial killing [20]. *Streptococcus pneumoniae* (pneumococcus) produces a peptidoglycan deacetylase (SpPgdA) and a *pgdA* gene deletion mutant showed increased susceptibility to lysozyme and reduced virulence in an intraperitoneal mouse model [21]. PGDAs have also been reported in other bacterial pathogens (reviewed in [22]). Another deacetylase has been reported in *Streptococcus iniae* and named polysaccharide deacetylase of *S. iniae* (SiPdi) [23]. The substrate for this enzyme is unknown, but a role in adherence and host invasion has been demonstrated. 

The *Staphylococcus epidermidis* deacetylase (IcaB) alters bacterial biofilms by modifying poly-β-1,6-N-acetyl-d-glucosamine (PNAG), also referred to as polysaccharide intercellular adhesin (PIA), an exopolysaccharide that forms the extracellular matrix of some bacterial biofilms [24]. Deletion of the *icaB* gene resulted in impaired biofilm formation and colonisation of the bacteria [25].

Recently, we reported the biochemical characterisation of two novel *S. pyogenes* deacetylases, Spy1370 and Spy1094 [26]. Recombinant forms of the two enzymes deacetylated the pseudosubstrate GlcNAc_3_, suggesting a role in the modification of the bacterial peptidoglycan cell wall. In this study, we investigated the involvement of Spy1094 and Spy1370 on *S. pyogenes* virulence by generating *S. pyogenes* gene deletion mutants and *Lactococcus lactis* gain-of-function mutants and analysed them for lysozyme sensitivity, antimicrobial resistance, biofilm formation, survival in human whole blood and in-vivo virulence using a *Galleria mellonella* (greater wax moth) larvae infection model. 

## 2. Materials and Methods

### 2.1. Bacterial Strains and Growth Conditions

*S. pyogenes* SF370 (M1 serotype, ATCC 700294) was grown in Brain Heart Infusion (BHI) (BD Biosciences, San Jose, CA, USA) at 37 °C under static conditions. *Lactococcus lactis* MG1363 was cultured in M17 + 0.5% glucose (GM17) medium (BD Biosciences) at 28 °C under static conditions. *Escherichia coli* DH5α was grown in Luria Bertani (LB, BD Biosciences) broth at 37 °C with aeration. Solid BHI, GM17 or LB plates were made by adding 1.5% Bacto agar (BD Biosciences) to the liquid medium. When appropriate, antibiotics were added: spectinomycin at 50 μg/mL for *S. pyogenes* and 100 μg/mL for *E. coli*; kanamycin at 200 μg/mL for *S. pyogenes* and *L. lactis*, and 50 μg/mL for *E. coli*.

### 2.2. Generation of S. pyogenes and L. lactis Mutants

*S. pyogenes Δspy1094* and *S. pyogenes Δspy1370* mutants were generated by allelic replacement using the pFW11 vector [27] (a gift from Andreas Podbielski, University of Rostock) as previously described [28,29]. In brief, ~1000 bp sequences flanking the upstream (FR1) and downstream (FR2) regions of the *spy1370* and *spy1094* genes, respectively, were amplified from genomic *S. pyogenes* DNA by 30 cycles of PCR with iProof polymerase (Bio Rad, Hercules, CA, USA) and specific primers (Sigma-Aldrich, St. Louis, MO, USA) (see Table 1). Each flanking region was cloned into the pFW11 MCS-I and MCS-II regions, respectively, which flank the spectinomycin-resistance gene *aad9*, using restriction enzymes (NEB, Table 1) and T4 DNA-ligase (Biolab, Tokyo, Japan). The recombinant constructs were electroporated into *S. pyogenes* SF370 using a Gene Pulser Xcell—(Bio-Rad), and the transformants were selected on BHI agar plates containing spectinomycin. Replacement of the *spy1094* or *spy1370* genes with *aad9* was confirmed by PCR using flanking region (FR) primers and *aad9*.fw primers (Table 1). In addition, the deletion mutants were validated at the protein level by Western blotting (see below). The *S. pyogenes* gene deletion mutants were complemented with the complete *spy1094* gene and *spy1370* gene, respectively, cloned into the pLZ12-Km2-P23R plasmid [27] using gene-specific primers (Table 1).

*L. lactis* gain-of-function strains were generated by electroporating the recombinant pLZ12-km2-P23R plasmids into *L. lactis*. The strains were validated by PCR and Western blotting.

### 2.3. Western Blots

Whole bacterial cell lysates were separated on a 12.5% polyacrylamide gel and transferred onto a nitrocellulose membrane (Bio Rad) in a transfer buffer (25 mM Tris-HCl, 192 mM glycine, 20% (*v*/*v*) methanol, pH 8.3) using a TE77 semi-transfer unit (Hoefer, Holliston, MA, USA) at 200 V, 50 mA/gel for 1 h. The membrane was incubated with TBS-T (20 mM Tris-HCl pH 7.6, 150 mM NaCl, 0.1% (*v*/*v*) Tween-20) plus 5% (*w*/*v*) skim milk powder at RT for 1 h and then washed once with TBS-T. Antibodies against recombinant forms of Spy1094 and Spy1370 were generated in rabbits as previously described [26]. Rabbit-sera were diluted 1/1000 with TBS-T plus 2.5% (*w*/*v*) milk powder and incubated with the membranes for 1 h at RT. After three washes with TBS-T, the membranes were incubated with HRP-cojugated goat anti-rabbit IgG (Abcam, Cambridge, UK), diluted 1/1000 in TBST for 1 h at RT. The membranes were then washed three times with TBS and developed using ECL detection reagent (Amersham Biosciences, Slough, UK) and analysed with a ChemiDoc^TM^ imaging system (Bio Rad).

### 2.4. Effects of Lysozyme on the Survival of S. pyogenes and L. lactis Strains

The protocol for lysozyme killing assays were adapted from Milani et al. [23]. Overnight cultures of *S. pyogenes* wildtype and mutant strains were grown in BHI with appropriate antibiotics to the mid exponential phase (OD_600_ = 0.6–0.8). Bacteria were then washed with PBS and resuspended in BHI to approximately 2 × 10^5^ CFU. An amount of 20 μL chicken egg white lysozyme (Sigma-Aldrich) was added to 180 μL of diluted bacteria to a final concentration of 80 μg/mL. The bacteria were then incubated at 37 °C for 30 min, serially diluted in sterile PBS and enumerated by spot-plating in triplicates onto BHI agar plates containing the appropriate antibiotics. Cultures without lysozyme treatment were grown as controls. Percentage survival was calculated using the following equation: % survival = ([CFU with treatment]/[CFU without treatment]) × 100. Four independent experiments were carried out.

### 2.5. Effects of Cecropin B on the Survival of GAS Mutants

This method was adopted from Wang et al. [27]. Overnight cultures of each *S. pyogenes* strain were grown to the mid exponential phase (OD_600_ = 0.6–0.8) at 37 °C in BHI with the appropriate antibiotics. Cecropin B (Sigma-Aldrich) was serially diluted (0–25 μM) and added to bacterial cultures in a final volume of 100 μL in a 96-well plate. The cultures were incubated further at 37 °C until the stationary phase was reached and then serially diluted for enumeration by spot-plating in triplicates. Cultures without cecropin B treatment were grown as controls. Percentage survival was calculated using the following equation: % survival = ([CFU with treatment]/[CFU without treatment]) × 100. Three independent experiments were carried out.

### 2.6. Effects of H_2_O_2_ on the Survival of GAS Mutants

This method was adopted from Pericone et al. [28]. Overnight cultures of each GAS strain were grown to the mid-exponential phase (OD_600_ = 0.6–0.8) at 37 °C, supplemented with the appropriate antibiotics). Then, 100 μL aliquots were added to 100 μL of fresh BHI media containing 0–5% H_2_O_2_. The samples were then incubated at 37 °C for 1 h before spot-plating in triplicates for bacterial enumeration. Control cultures without H_2_O_2_ treatment were grown in parallel. Percentage survival was calculated using the following equation: % survival = ([CFU with treatment]/[CFU without treatment]) × 100. Four independent experiments were carried out.

### 2.7. Biofilm Formation in GAS Mutants

*S. pyogenes* wildtype and mutant strains were grown at 37 °C overnight and then diluted 1:10 in fresh BHI media containing the appropriate antibiotics. The cultures were then seeded into uncoated 96-well plates (Sigma-Aldrich) in triplicates and further incubated at 37 °C overnight without aeration. Unbound, planktonic cells were removed by washing the wells with sterile PBS before the addition of crystal violet dye as described previously [29]. Biofilm formation was quantified by measuring absorbance at 595 nm using an EnSightTM Multimode plate reader (Perkin Elmer, Waltham, MA, USA). Four independent experiments were carried out.

### 2.8. Whole Blood Killing Assay

This assay was carried out as described recently [30]. *S. pyogenes* wildtype and mutant strains were grown in BHI with appropriate antibiotics to the late exponential phase. Approximately 1 × 10^5^ CFU of bacteria in a volume of 50 μL were added to 1 mL of fresh heparinised human whole blood from a consented donor and incubated for 2.5 h at 37 °C with constant rotation. An amount of 100 μL samples were taken every 30 min, serially diluted and plated onto BHI agar plates in triplicates. The percentage of survival was calculated as [CFU (at a given time point)/CFU (at the start)] × 100.

*L. lactis* was grown in a GM17 medium with appropriate antibiotics to the late exponential phase, pelleted at 5000× g and resuspended in Hank’s Balanced Salt Solution (HBSS). Approximately 1 × 10^3^ or 1 × 10^5^ bacteria were added to 1 ml of heparinised human blood and incubated for 2.5 h at 37 °C. Serially diluted samples were plated on GM17 agar plates in triplicates. Bacterial samples were also cultured on agar plates and enumerated to confirm the injected doses. Two independent experiments with two different blood donors were carried out.

Collection of human blood was approved by the Auckland Health Research Ethics Committee (AH24859) for a project entitled “Investigation of host-pathogen interactions to identify new targets and develop new detection/diagnostic tools for anti-microbial therapy”. Volunteers without a previous episode of pharyngitis/tonsillitis were informed that their blood would be used for research only, assigned a code and de-identified by the phlebotomist. No blood samples were stored after the assays.

### 2.9. Galleria Mellonella Larvae Infection Model

*G. mellonella* (greater wax moth) larvae experiments were carried out as previously described [31,32]. In brief, a group of 10 antibiotic-free, healthy (with no melanisation) and medium-sized (~1.5 cm) *G. mellonella* larvae (Biosuppliers-New Zealand) were used for each experiment. Twenty microlitres of bacteria (1.5 × 10^6^ CFU for *S. pyogenes* wildtype and mutant strains and 1 × 10^5^ CFU for *L. lactis* wildtype and mutant strains) were injected into the lower left proleg using an insulin syringe (BD Biosciences). A control group was injected with sterile PBS only. Bacterial samples were also cultured on agar plates and enumerated to confirm the injected doses. The larvae were incubated in Falcon^®^12-well plates (Biocompare) at 37 °C without food. Survival of the larvae was monitored over a 5-day period post-injection. In addition, a health index score was calculated as described previously [31,32]. 

### 2.10. Statistical Analysis

All statistical analyses were conducted using GraphPad Prism software (version V7.03). Statistical significance was calculated using one-way ANOVA with Tukey’s multiple comparisons to compare two or more data sets. *p* values of <0.05 were considered statistically significant. Survival curves were estimated using the Kaplan–Meier estimator in GraphPad Prism.

## 3. Results

### 3.1. Generation of S. pyogenes Gene Deletion Strain and L. lactis Gain-of-Function Strains

**Figure 1:**
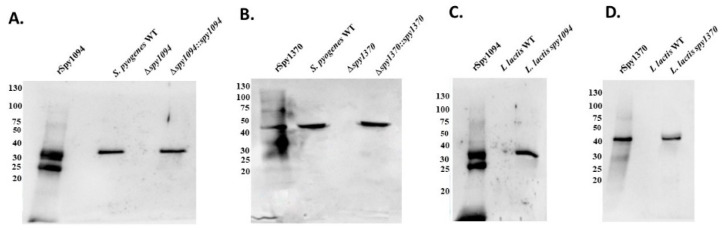
Expression of Spy1094 and Spy1370 in wild-type and mutant strains. Bacterial whole cell lysates were separated on a 12.5% PAGE gel, transferred onto a nitrocellulose membrane and probed with rabbit antiserum against rSpy1094 or rSpy1370. (**A**) Expression of Spy1094 in wildtype and mutant *S. pyogenes.* (**B**) Expression of Spy1370 in wildtype and mutant *S. pyogenes*. (**C**) Expression of Spy1094 in wildtype and mutant *L. lactis.* (**D**) Expression of Spy1370 in wildtype and mutant *L. lactis*. Recombinant Spy1094 or rSpy1370 were used as positive control.

We have previously shown that recombinant forms of Spy1094 and Spy1370 possess deacetylase activity for the pseudosubstrate GlcNAc_3_ which is commonly used to demonstrate the ability to deacetylase peptidoglycan cell walls [26]. To demonstrate that the two enzymes play a crucial role in *S. pyogenes* virulence, we first generated isogenic gene deletion mutants. Allelic replacement was used to exchange the target gene for the spectinomycin-resistance gene *aad9*. The deletion mutants were then complemented with the respective complete genes driven by the constitutive lactococcal P23 promotor [33]. The successful gene deletion was confirmed by Western blot analysis with specific antisera raised against recombinant forms of the enzymes (Figure 1A). Bands were detected in the wildtype strain at ~32 kDa (Spy1094) and ~44 kDa (Spy1370) which correspond to the correct sizes of the enzymes as determined previously [26]. Those bands were not detected in the deletion mutants but were visible in the complementation mutants (Figure 1A,B). 

The complementation plasmids were also used to generate gain-of-function mutants in *L. lactis*. As shown in Figure 1C,D, the specific antisera did not react with any protein in the wildtype *L. lactis* whole cell lysate, but recognised proteins at the expected sizes in the extracts of the gain-of-function mutants, confirming expression of Spy1094 and Spy1370. 

**Figure 2:**
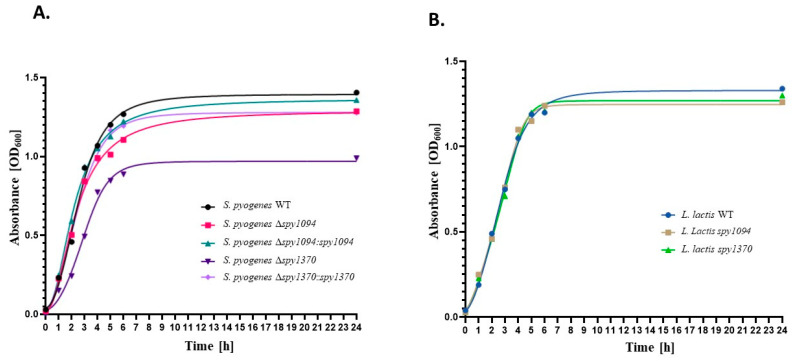
In-vitro growth kinetics of wildtype and mutant strains. Overnight cultures of wildtype and mutant strains were adjusted to an OD_600_ of 0.04 and grown for 24 h at 37 °C. (**A**) *S. pyogenes* strains. (**B**) *L. lactis* strains.

Growth kinetics were conducted with wildtype and mutant strains. The *S. pyogenes Δspy1094* mutant showed a slightly decreased growth compared to the parent strain, whereas *S. pyogenes Δspy1370* grew considerably slower and failed to reach the same cell density as the wildtype strain after 24 h (Figure 2A). Complementation of the mutant strains resulted in growth kinetics similar to the *S. pyogenes* wildtype, suggesting that the growth defects could be solely attributed to the deleted genes. No difference in growth was observed between the *L. lactis* mutants compared to the parent strain (Figure 2B).

### 3.2. Deletion of spy1094 and spy1370 Decreases Lysozyme Resistance

**Figure 3:**
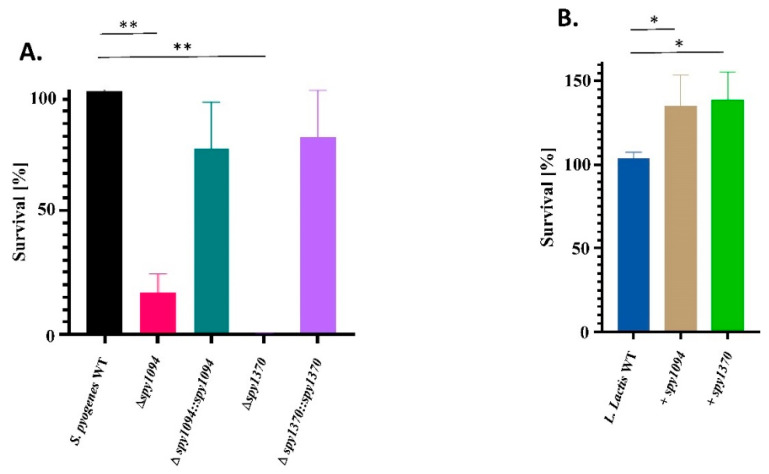
Spy1094 and Spy1370 contribute to lysozyme resistance. Wildtype and mutant strains were treated with 80 μg/mL lysozyme and incubated for 30 min at 37 °C. Surviving bacteria were enumerated by spot plating in triplicates on agar plates. (**A**) *S. pyogenes* strains. (**B**) *L. lactis* strains. Data are mean values from four independent experiments in triplicates +/− SD. *p* values were calculated using the one-way ANOVA tests. ** *p* < 0.001; * *p* < 0.05.

There is a direct relationship between increasing levels of deacetylation of the peptidoglycan cell wall and increasing resistance to lysozyme [34]. We previously showed that recombinant forms of Spy1094 and Spy1370 both deacetylate the pseudosubstrate GlcNAc_3_, suggesting a biological role in peptidoglycan modification [26]. To further analyse this, we treated wildtype and mutant strains with 80 μg/mL lysozyme for 30 min and enumerated the surviving bacteria. As shown in Figure 3A, deletion of the *spy1094* gene led to an ~85% reduction of survival (*p* < 0.001), whereas the *S. pyogenes Δspy1370* mutant was completely killed by the lysozyme treatment. Complementation of the genes restored lysozyme resistance to approximately 75% (*spy1094*) and ~80% (*spy1370*) of wildtype *S. pyogenes*. Heterologous expression of Spy1094 and Spy1370 in *L. lactis* led to a ~35% increase in lysozyme resistance (*p* < 0.05) (Figure 3B). 

### 3.3. Deletion of spy1370 Confers Resistance to Cationic AMP, but Not to Oxidative Killing

Deacetylation of the peptidoglycan cell wall leads to an increase in the net positive charge which might affect the activity of cationic antimicrobial peptides (CAMPs) by preventing translocation to the cell membrane. It has been demonstrated that deletion of the *Staphylococcus epidermidis icaB* gene, which encodes an exopolysaccharide deacetylase, results in decreased resistance against CAMPs and neutrophil phagocytosis [35]. We, therefore, analysed our wildtype and mutant strains for resistance to Cecropin B, a CAMP that was first isolated from the hemolymph of the silk moth *Hyalophora cecropia* [36] and to H_2_O_2_, a reactive oxygen species produced inside phagosomes during the oxidative burst. 

**Figure 4:**
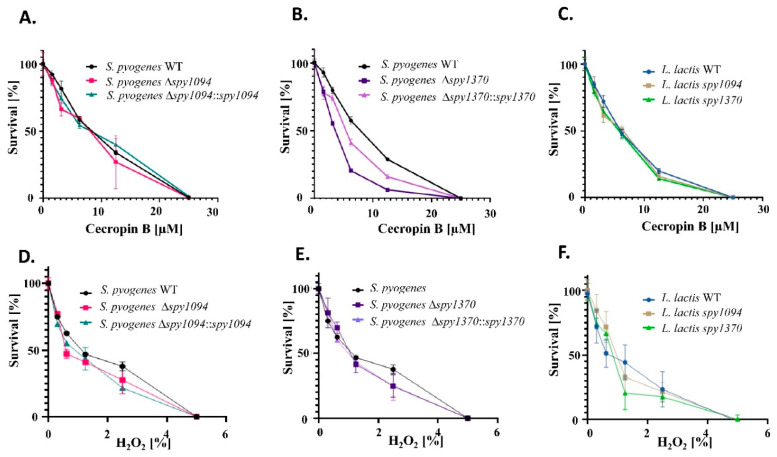
Survival of wild-type and mutant strains after treatment with the CAMP cecropin B or with H_2_O_2_. Wildtype and mutant strains were grown to mid-exponential phase (OD_600_ = 0.6–0.8), treated with cecropin B at various concentrations (0–25 μM) and incubated for another 3 h at 37 °C. (**A,B**) *S. pyogenes* wildtype and mutants. (**C**) *L. lactis* wild-type and mutants. To measure susceptibility to oxidative killing, bacteria were treated with H_2_O_2_ at concentrations of 0–5% and incubated for another 30 min at 37 °C. (**D,E**) *S. pyogenes* wildtype and mutants. (**F**) *L. lactis* wild-type and mutants. Surviving bacteria were enumerated by spot plating in triplicates on agarose. Data are mean values from two independent experiments (**A**–**C**) or four independent experiments (**D**–**F**) in triplicates +/− SD. Notably, the *S. pyogenes Δspy1370* mutant showed decreased growth in culture medium which could be restored in a complementation mutant (Figure 2A).

Cecropin B showed a dose-dependent effect on *S. pyogenes* and *L. lactis* with complete bacterial killing observed at concentrations of 25 μM (Figure 4A–C). Deletion of *spy1370* resulted in substantially decreased survival at the concentrations tested (*p* < 0.001). This was partially restored in the *S. pyogenes Δspy1370::spy1370* complementation mutant (Figure 4B). No differences were observed between the *spy1094* deletion mutant and wildtype *S. pyogenes* (Figure 4A) or between wildtype *L. lactis* and the gain-of-function mutants (Figure 4C). The effect of H_2_O_2_ was measured at various concentrations up to 5%, which resulted in the complete killing of the bacteria (Figure 4D–F). No significant differences were observed between wildtype bacteria and mutant strains, suggesting that Spy1094 and Spy1370 have no protective effect against exogenous H_2_O_2_.

### 3.4. Deletion of spy1094 and spy1370 Decreases Biofilm Formation

Biofilms are responsible for a large medical burden on a global scale. *S. pyogenes* is able to form biofilms which have been associated with diseases such as tonsilitis and cellulitis [37]. Recently, it was shown that *S. pyogenes* serotype M1 strains consistently formed biofilms in patients with necrotising fasciitis (‘flesh-eating disease’) [38]. 

**Figure 5:**
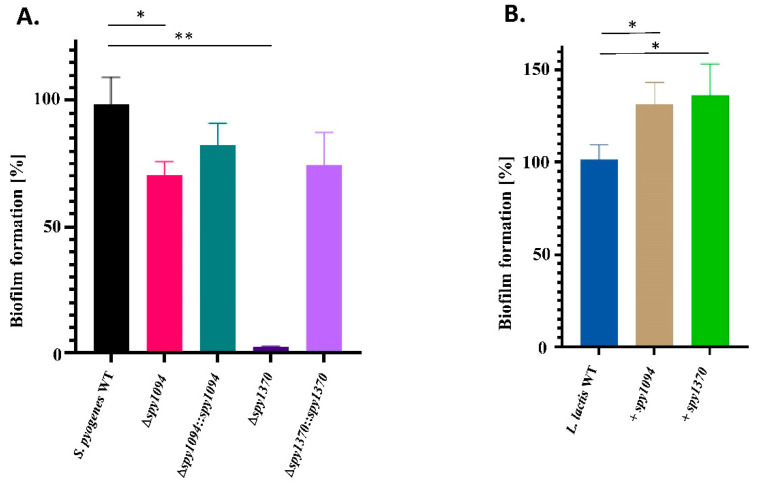
Spy1094 and Spy1370 contribute to biofilm formation. Wildtype and mutant strains were grown on uncoated 96-well plates and incubated at 37 °C overnight. Biofilms were measured in triplicates after addition of crystal violet dye by measuring absorbance at 595 nm. (**A**) *S. pyogenes* wildtype and mutants. (**B**) *L. lactis* wild-type and mutants. Data are mean values from four independent experiments in triplicates +/− SD. *p* values were calculated using the one-way ANOVA tests. ** *p* < 0.001; * *p* < 0.05.

As the *S. epidermitis* exopolysaccharide deacetylase IcaB plays an important role in biofilm formation, we analysed the ability of Spy1094 and Spy1370 to contribute to biofilm formation in-vitro. Biofilm formation was observed with the wildtype *S. pyogenes* SF370 strain (serotype M1) but was significantly reduced to <5% in the *spy1370* deletion mutant (*p* < 0.001) (Figure 5A). The *S. pyogenes Δspy1370::spy1370* complementation mutant restored biofilm formation to approximately 75%. Deletion of *spy1094* also resulted in significantly decreased biofilm formation (*p* < 0.05) to ~70% of the wildtype level and was also partly restored in the complementation mutant to 80% (Figure 5A). These findings could be confirmed with the *L. lactis* gain-of-function mutants where the *L. lactis spy1094* and *L. lactis spy1370* mutants showed increased biofilm formation (*p* < 0.001) compared to *L. lactis* wildtype at levels of 140% and 150%, respectively (Figure 5B).

### 3.5. Spy1094 and Spy1370 Promote Survival in Human Blood

**Figure 6:**
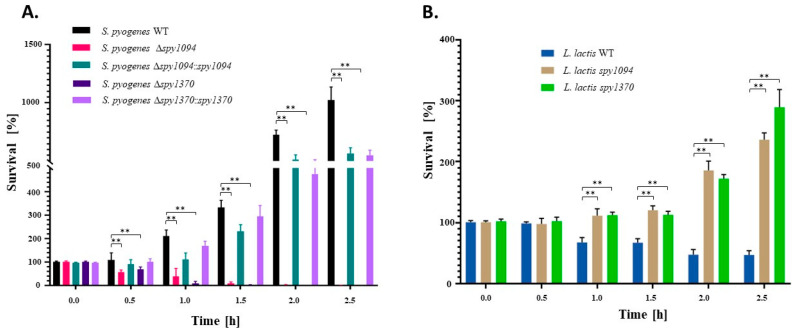
Survival of wildtype and mutant bacteria in whole human blood. Freshly collected human blood was inoculated with 1 × 10^5^ CFU per ml with (**A**) *S. pyogenes* and mutant strains or (**B**) with *L. lactis* and mutant strains. Surviving bacteria were enumerated by spot-plating in triplicates on BHI and GM17 agar plates, respectively. Data are mean values from two independent experiments with two different blood donors in triplicates +/− SD for *S. pyogenes* (**A**). For *L. lactis,* one representative experiment in triplicates is shown and a second experiment with a different blood donor and a lower inoculation (1 × 10^3^ CFU) is shown in supplementary Figure S1. *p* values were calculated using the one-way ANOVA tests. ** *p* < 0.001.

To test if the two deacetylases contribute to the overall virulence of *S. pyogenes*, we used a human whole blood killing assay [30]. Freshly collected human blood was inoculated with 1 × 10^5^ CFU *S. pyogenes* wildtype and mutant strains, and survival was analysed by enumerating the surviving bacteria (Figure 6A). The *S. pyogenes* wildtype showed about a 10-fold increase in CFU during the 2.5 h incubation period. In contrast, survival of *S. pyogenes Δspy1094* and *S. pyogenes Δspy1370* both significantly decreased during incubation and were almost completely killed within the first 1.5 h (*p* < 0.001; Figure 6A). A difference between the mutant strains was observed after 1 h when approximately 50% of the *S. pyogenes Δspy1094* still survived, whereas *S. pyogenes Δspy1370* was almost completely killed. Survival in blood was mostly restored in the complemented mutants. We also analysed *L. lactis* wildtype and mutant strains (Figure 6B) by inoculating human blood with 1 × 10^5^ CFU bacteria and found that both mutants survived in whole human blood with a ~2.2-fold increase in CFU for *L. lactis spy1094* and ~3-fold increase in CFU for *L. lactis spy1370* at the 2.5 h endpoint. In contrast, <50% of the *L. lactis* wildtype survived after the 2.5 h incubation showing that the two deacetylases significantly increase survival of *L. lactis* in human blood (*p* < 0.001). Similar results were achieved when human blood was inoculated with 1 × 10^3^ CFU of the *L. lactis* wildtype or mutant strains (Supplementary Figure S1).

### 3.6. Deletion of spy1094 and spy1370 Reduces Virulence of S. pyogenes in a Galleria Mellonella Infection Model

*G. mellonella* (wax moth) larvae have been introduced as an alternative model to study microbial infections and we have previously shown that this model is useful to study virulence in *S. pyogenes* [31,32,39]. Although insects lack an adaptive immune response, their innate immune response shows remarkable similarities with the immune response in vertebrates, including the presence of lysozyme and AMPs [39]. 

**Figure 7:**
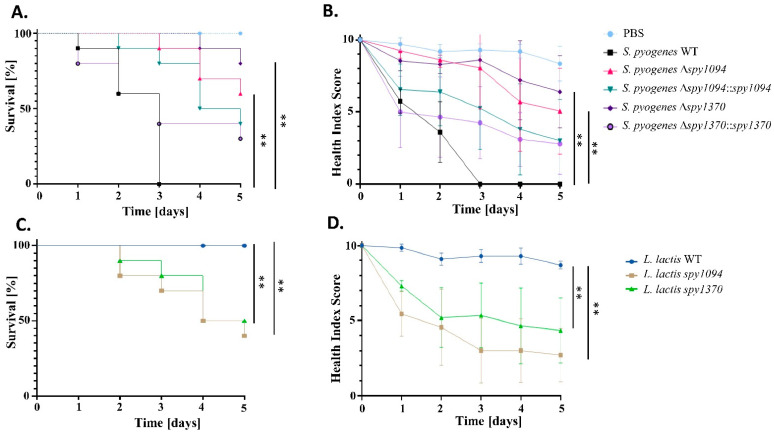
Infection of *G. mellonella* larvae shows a role for Spy1094 and Spy1370 in *S. pyogenes* virulence. Twenty microlitres of bacteria (1.5 × 10^6^ CFU for *S. pyogenes* wildtype and mutant strains and 1 × 10^5^ CFU for *L. lactis* wildtype and mutant strains) were injected into the lower left proleg of *G. mellonella* larvae (*n* = 10). A control group was injected with sterile PBS only. Survival of the larvae was monitored over a 5-day period post-injection. (**A**) *S. pyogenes* wildtype and mutants. (**C**) *L. lactis* wild-type and mutants. In addition, a health index score was calculated which includes mobility, cocoon formation and melanisation of the infected larvae. (**B**) *S. pyogenes* wildtype and mutants. (**D**) *L. lactis* wild-type and mutants. *p* values were calculated using the one-way ANOVA tests. ** *p* < 0.001.

Compared to infection with wildtype *S. pyogenes*, deletion of *spy1094* or *spy1370* substantially increased survival of infected *G. mellonella* larvae (Figure 7A). Infection with wildtype *S. pyogenes* resulted in the complete killing of the larvae after three days. In contrast, only 10% killing was observed after three days with the *S. pyogenes Δspy1094* mutant and 60% of the larvae were still alive after five days. The complementation mutant partially restored the wildtype phenotype with larval survival reduced to 40% after five days. An even larger effect was observed with the *S. pyogenes Δspy1370* mutant which did not kill any larvae three days post-infection and 80% of the larvae were still alive after five days. Only 30% larval survival five days post-infection was observed with the complementation mutant. We have previously developed a health score index which allows assessment of the *G. mellonella* larvae not only based on survival, but also certain health indicators such as melanisation, cocoon formation and mobility [31,39]. On a scale from 1 to 10 with 10 being the most healthy larvae, larvae infected with the *S. pyogenes spy1094* and *spy1370* deletion mutants both showed substantially increased health scores. Larvae inoculated with either *S. pyogenes Δspy1094* or *S. pyogenes Δspy1370* both scored about 9 after the first three days post-infection, dropping to 7 after five days. In contrast, larvae infected with wildtype *S. pyogenes* scored only 0.5 three days post-infection (Figure 7B). Infection of *G. mellonella* larvae with the *L. lactis* gain-of-function mutants further confirmed the role of Spy1094 and Spy1370 in *S. pyogenes* virulence. *L. lactis* expressing Spy1094 killed 60% of the larvae after five days compared to the 100% survival of larvae infected with wildtype *L. lactis*. The *L. lactis spy1370* mutant killed 50% of larvae after five days (Figure 7C). For the health score index, larvae inoculated with the *L. lactis* mutants expressing Spy1094 or Spy1370 scored 5 two days post-infection. After five days, the scores were 3 and 5, respectively. In contrast, the health score in larvae inoculation with wildtype *L. lactis* dropped only slightly to 9 after five days (Figure 7D).

## 4. Discussion

We have recently reported the biochemical analysis of the *S. pyogenes* deacetylases Spy1094 and Spy1370. Recombinant forms of both enzymes were able to deacetylate the pseudosubstrate chitotriose (GlcNAc_3_) with high substrate affinity but low substrate turnover. Here, we describe the further characterisation of the enzymes using gene deletions introduced by allelic replacement with the spectinomycin-resistance gene *aad9*. In addition, *L. lactis* gain-of-function mutants were generated. Our results suggest that both enzymes deacetylate the peptidoglycan cell wall, contributing to lysozyme resistance and increased virulence during infection. 

Peptidoglycan GlcNAc deacetylases (PgdA enzymes) have been described in several pathological bacterial species, mainly in Gram-positive bacteria. The first description of a PgdA enzyme was reported in 2000 when it was shown that deletion of the *pgdA* gene in *Streptococcus pneumoniae* resulted in increased susceptibility to lysozyme [40]. Lysozyme is an enzyme that is abundantly found in mucosal secretions, including tears and saliva. Lysozyme hydrolyses the β-1,4-glycosidic bonds between MurNAc and GlcNAc and plays an important role in innate immunity [41]. PgdA enzymes have also been reported in the swine pathogen *Streptococcus suis* and in *Streptococcus mutans* but play no role in lysozyme resistance despite the fact that SmPgdA is able to deacetylate chitohexaose (GlnNAc_6_) [42,43]. The fish pathogen *Streptococcus iniae* expresses a paralogue deacetylase (Pdi) with unknown substrate specificity. However, a *pdi* gene deletion revealed a role for Pdi in lysozyme resistance [23]. The *S. pyogenes* serotype M1 Spy1370 is 35% identical to PgdA from *S. pneumoniae*, whereas Spy1094 shares 72% identity with Pdi from *S. iniae*, suggesting orthologue genes [26]. In line with the results reported for *S. pneumoniae* PgdA, *spy1370* gene deletion resulted in significantly decreased lysozyme resistance (*p* < 0.001). More recently, a Spy1094 variant was characterised in the *S. pyogenes* NZ131 strain (serotype M49) and named polysaccharide-peptidoglycan linkage deacetylase (PplD). The authors found that PplD deacetylates the GlcNAc that is linked with the rhamnopolysaccharide of the Group A antigen [44]. Notably, the study shows no decrease in lysozyme resistance after deletion of the *pplD* gene, which is in contrast to Pdi [23] and our results, which show a significant decrease in lysozyme resistance in the *spy1094* deletion mutant (*p* < 0.001). A possible reason might be the growth phase of the bacteria used in the lysozyme assays. It was shown that *S. iniae Δpdi* mutants were substantially more susceptible to lysozyme when static bacteria were exposed compared to growing cells [23]. In our assay, the bacteria were also used at static conditions. We also provide further evidence for the role of Spy1094 as a PgdA-like enzyme by generating *L. lactis* gain-of-function mutants. *L. lactis* is a non-pathogenic bacterium that expresses a PgdA enzyme that provides resistance to autolysis. It was also shown that overexpression of PdgA results in a higher percentage of deacetylated peptidoglycan [45]. Our results indicate that expression of either Spy1094 or Spy1370 in *L. lactis* leads to a significant increase in lysozyme resistance (*p* < 0.05). This suggests that either PplD/Spy1094 deacetylates other GlcNAc residues in the cell wall and, therefore, has at least a partly overlapping function with PgdA or that deacetylation of the cell wall linkage unit is sufficient to confer some resistance to lysozyme. 

Notably, lysozyme can kill bacteria due to a non-enzymatic mechanism similar to cationic antimicrobial peptides (CAMPs) that requires a negatively charged cell wall which facilitates the transport across the cell wall [41]. Deacetylation of GlcNAc increases the cell wall net positive charge which might lead to repulsion of CAMPs. Cecropin B is a CAMP that was shown to be highly effective against *E. coli* and the Gram-positive *Staphylococcus aureus* [27]. Our results show that Spy1370, but not Spy1094, confers resistance to cecropin B (*p* < 0.001) which is in line with the results reported for *S. iniae* where neither moronecidine nor polymyxin B showed increased killing in the *pdi* gene deletion mutant [23]. In contrast, *S. pyogenes* NZ131 strains with either *pgdA* or *pplD* gene deletions showed reduced resistance to killing by the cationic enzyme human group IIA secreted phospholipase A2 (hGIIA), which might be due to the very high positive charge of hGIIA [44]. 

Deacetylation of peptidoglycan might also reduce recognition by pattern-recognition receptors (PRRs) such as Toll-like receptor 2 (TLR-2) [46]. Furthermore, TLR-signalling results in the production of AMPs [41]. We provided further evidence for a role of GlcNAc deacetylases in immune modulation using a human whole blood killing assay. The decreased survival of the *S. pyogenes Δspy1094* and *S. pyogenes Δspy1370* mutants, as well as the increased survival of *L. lactis* bacteria expressing Spy1094 or Spy1370 suggests an immune evasion strategy conferred by the deacetylases. This might be mediated by the increased positive cell surface charge due to cell wall deacetylation and consequently, the repulsion of CAMPs as discussed earlier. However, additional other mechanisms can’t be excluded. Our results are further supported by a transcriptome analysis of *S. pyogenes* grown in human whole blood compared to bacteria grown in a growth medium. After 30 min, the *spy1370* transcript was upregulated a massive 1541-fold, whereas *spy1094* mRNA was increased 146-fold, suggesting major roles in virulence [47]. Milani et al. reported an approximately 50% reduced survival for the *S. iniae pdi* deletion mutant after 1 h in whole fish blood and suggested that this might be due to higher susceptibility to oxidative killing, but this could not be confirmed experimentally [23]. This is in line with our observations as deletion of either *spy1094* or *spy1370* failed to increase killing of *S. pyogenes* by hydrogen peroxide. A longitudinal transcriptome analysis of *S. pyogenes* in an experimental rhesus macaque pharyngitis model provided some evidence for the involvement of Spy1370 in inflammation. The regulation of *spy1370* mRNA transcription strongly correlated with C-reactive protein levels which was used as a phenotypic marker for inflammation. No differential regulation for *spy1094* was reported [48]. 

The attenuated virulence of *S. pyogenes Δspy1094* and *S. pyogenes Δspy1370* was further evidenced in a *G. mellonella* larvae infection model. Our results show that both *S. pyogenes* deletion mutants had a significantly lower larvae killing effect compared to wildtype *S. pyogenes* (*p* < 0.001). In case of the *spy1370* deletion mutant, this might partially be due to a lower growth kinetic that we have observed in-vitro. However, both *L. lactis* gain-of-function mutants showed a significantly increased ability to kill *G. mellonella* larvae (*p* < 0.001), and showed a comparable in-vitro growth kinetic with wildtype *L. lactis*. This is in line with a study showing decreased larval killing with the *S. iniae pdi* deletion mutant [23]. The reason for the reduced growth of *S. pyogenes Δspy1370* is unclear. The complete restoration of the wildtype phenotype in the complementation mutant indicates that the enzymatic activity of Spy1370 is responsible. Spy1370 might be a promiscuous enzyme that also recognises a substrate that is involved in cell growth regulation. An alternative role for PdgA has been suggested for the swine pathogen *S. suis*, which contains peptidoglycan with low levels of deacetylated components and is lysozyme sensitive. However, deletion of the *pdgA* gene resulted in severely impaired virulence in animal infection models [43]. Alternatively, peptidoglycan deacetylation might have an effect on cell wall morphology causing reduced growth. Notably, this reduced growth has not been reported for PdgA enzymes from any other species, but this might simply have been unrecognised. Interestingly, an *S. iniae* mutant lacking the *pdi* gene was unable to form chains and showed increased buoyancy in a liquid culture [23]. A similar defect was reported in the *L. lactis pdgA* deletion mutant [45]. However, we did not observe any differences in buoyancy or chain formation in *S. pyogenes Δspy1370* (data not shown).

We demonstrate that both Spy1094 and Spy1370 play a role in biofilm formation which was significantly decreased in the gene deletion mutants (*p* > 0.001) and increased in *L*. *lactis* gain-of-function mutants (*p* < 0.05). Involvement of PgdA deacetylases or other cell wall deacetylases in biofilm formation has not been reported in the literature thus far. However, Freiberg et al. reported a 3.7-fold upregulation of *spy1094* mRNA when *S. pyogenes* was grown in biofilm compared to planktonic growth at the stationary phase [49]. *Staphylococcus epidermitis* produces a deacetylase, IcaB, which targets polysaccharide intercellular adhesin (PIA), an important component of many bacterial biofilms. IcaB also deacetylates GlnNAc components. However, in PIA, these are β-1,6-linked and the enzyme lacks activity against β-1,4-linked GlnNAcs found in the peptidoglycan of the cell wall [35]. It, therefore, seems more likely that the deacetylation of the peptidoglycan GlcNAc and the resulting increase of the positive net charge might contribute to biofilm formation, e.g., by increasing bacterial cell aggregation. Notably, the *S. iniae pdi* deletion mutant had an approximately two-fold reduced ability to adhere to fish epithelial cells, and deletion of the *pdgA* gene in *S. mutans* resulted in a different colony texture and increased cell surface hydrophobicity [42].

## 5. Conclusions

We have generated *spy1094* and *spy1370* gene deletions in *S. pyogenes* SF370 (serotype M1) and gain-of-function mutants in *L. lactis*. Using these strains, we show that Spy1094/PplD and Spy1370/PgdA play crucial roles in *S. pyogenes* virulence in a human blood killing assay and a *G. mellonella* infection model. Both proteins are cell wall deacetylases that target GlcNAc components and contribute to lysozyme and biofilm formation. Furthermore, Spy1370/PgdA, but not Spy1094/PlpD, confers resistance to the cationic antimicrobial peptide cecropin B. Our results might provide a basis for the future development of specific treatment options against *S. pyogenes*.

## Figures and Tables

**Table 1:** Primers used in this study. The restriction enzyme sites are underlined.

**A: Primers Used to Generate and Confirm *S. pyogenes*** ***Δ*** ***spy1094* and *S. pyogenes*** ***Δ*** ***spy1370***		
**Primer**	**Sequence (5′-3′)**	**Restriction enzyme**
*spy1094*FR1.fw	tgcctcgagtcgagctgacgggttttc	XhoI
*spy1094*FR1.rev	aggtaagcttgaaaatgagggtcaaaccaa	HindIII
*spy1094*FR2.fw	agctgcagccaaatcatactcactgtaaac	PstI
*spy1094*FR2.rev	agcccgggttcagttcaagacctgttgac	XmaI
*spy1370*FR1.fw	cggtcgaccagttggtttagttcttgcc	SalI
*spy1370*FR1.rev	cgggatccaataaacacaatagctaaac	BamHI
*spy1370*FR2.fw	agctgcaggtaatatcgttatgtttc	PstI
*spy1370*FR2.rev	atacccgggcttagcttatgtctttccta	XmaI
*aad9*.fw	ccttattggtacttacatgtttg	none
*aad9*.rev	ccattcaatattctctccaag	none
**B: Primers used to generate *L. lactis*** ***spy1094* and *L. lactis*** ***spy1370***		
**Primer**	**Sequence (5′-3′)**	**Restriction enzyme**
*spy1094*RBS.fw	tcaggatccgattggagcaaataaatatgaacaatagacataaacggc	BamHI
*spy1094.rev*	gaattcttatggttccattgtttg	EcoRI
*spy1370*RBS.fw	tcaggatccgattggagcaaataaatatgaaaaaattaaatgttattcttgttg	BamHI
*spy1370*.rev	ccgctcgagttactgatgcgcatagag	XhoI

## Data Availability

Data is contained within the article and in Supplementary Figure S1.

## Supplementary Materials

The following supporting information can be downloaded at: https://www.mdpi.com/article/10.3390/microorganisms11020305/s1, Figure S1: Survival of wildtype and mutant *L. lactis* strains in whole human blood after incubation with 1 × 10^3^ CFU.
