# Supplementary material for: The Cell Wall Deacetylases Spy1094 and Spy1370 Contribute to Streptococcus pyogenes Virulence
Source: Microorganisms. 2023 Jan 24;11(2):305. doi: 10.3390/microorganisms11020305 (PMC9966966; doi:10.3390/microorganisms11020305)
Supplement: Supplementary file 1 [file microorganisms-11-00305-s001.zip › microorganisms-2087829-supplementary.pdf]

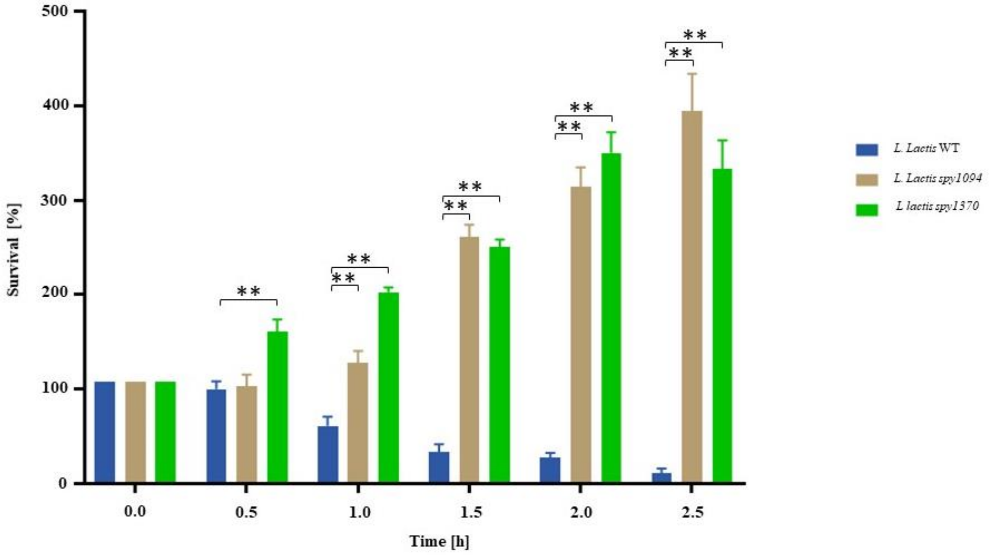

Supplementary Figure S1. Survival of wildtype and mutant *L. Lactis* strains in whole human blood

Freshly collected human blood was inoculated with 1 x 10^3 CFU per ml with *L. lactis* and mutant strains. Surviving bacteria were enumerated by spot-plating in triplicates on GM17 agar plates. Data are mean values +/- SD. *P* values were calculated using the one-way ANOVA tests. ***P*<0.001.
